# Ab initio and homology based prediction of protein domains by recursive neural networks

**DOI:** 10.1186/1471-2105-10-195

**Published:** 2009-06-26

**Authors:** Ian Walsh, Alberto JM Martin, Catherine Mooney, Enrico Rubagotti, Alessandro Vullo, Gianluca Pollastri

**Affiliations:** 1 School of Computer Science and Informatics, University College Dublin, Belfield, Dublin 4, Ireland; 2 Complex and Adaptive Systems Laboratory, University College Dublin, Belfield, Dublin 4, Ireland

## Abstract

**Background:**

Proteins, especially larger ones, are often composed of individual evolutionary units, domains, which have their own function and structural fold. Predicting domains is an important intermediate step in protein analyses, including the prediction of protein structures.

**Results:**

We describe novel systems for the prediction of protein domain boundaries powered by Recursive Neural Networks. The systems rely on a combination of primary sequence and evolutionary information, predictions of structural features such as secondary structure, solvent accessibility and residue contact maps, and structural templates, both annotated for domains (from the SCOP dataset) and unannotated (from the PDB). We gauge the contribution of contact maps, and PDB and SCOP templates independently and for different ranges of template quality. We find that accurately predicted contact maps are informative for the prediction of domain boundaries, while the same is not true for contact maps predicted ab initio. We also find that gap information from PDB templates is informative, but, not surprisingly, less than SCOP annotations. We test both systems trained on templates of all qualities, and systems trained only on templates of marginal similarity to the query (less than 25% sequence identity). While the first batch of systems produces near perfect predictions in the presence of fair to good templates, the second batch outperforms or match ab initio predictors down to essentially any level of template quality.

We test all systems in 5-fold cross-validation on a large non-redundant set of multi-domain and single domain proteins. The final predictors are state-of-the-art, with a template-less prediction boundary recall of 50.8% (precision 38.7%) within ± 20 residues and a single domain recall of 80.3% (precision 78.1%). The SCOP-based predictors achieve a boundary recall of 74% (precision 77.1%) again within ± 20 residues, and classify single domain proteins as such in over 85% of cases, when we allow a mix of bad and good quality templates. If we only allow marginal templates (max 25% sequence identity to the query) the scores remain high, with boundary recall and precision of 59% and 66.3%, and 80% of all single domain proteins predicted correctly.

**Conclusion:**

The systems presented here may prove useful in large-scale annotation of protein domains in proteins of unknown structure. The methods are available as public web servers at the address:  and we plan on running them on a multi-genomic scale and make the results public in the near future.

## Background

Proteins, especially larger ones, are often composed of individual evolutionary units, domains, which have their own function and structural fold. Predicting domains is an important intermediate step in protein analyses, including the prediction of protein structures. In this case the prediction can be applied to each protein domain separately, decreasing prediction times, and increasing prediction accuracy especially in the absence of homologues/templates and when interactions among residues are long ranging. Although domain-domain interactions would have to be ignored when predicting domain structures separately, stages for domain-domain interaction prediction can be designed [1,2] to tie the domains together resulting in the final three dimensional (3D) structure. The detection of structural templates from sequence can also be improved when only considering the sequence that corresponds to each domain, since the domain itself is more likely to be evolutionarily conserved. Fold recognition methods also perform better when using individual domains rather than the entire protein [3].

Experimental structural determination methods become hard to apply when considering large proteins of many domains. In X-Ray crystallography and NMR spectroscopy difficulties often arise when protein domains are joined by less flexible boundary regions. Also, NMR structural determination errors tend to arise when the protein is very long. As a result, experimental methods often determine structures by only examining individual domains or at most a few domains together [4,5].

Methods for the prediction of protein domains, similarly to methods for the prediction of the 3D structure, can be classified as template-based or template-free (which we will refer to as "ab initio"), depending on whether the prediction incorporates structural information from putative homologues from the Protein Data Bank [6]. The simplest form of domain prediction assumes all domains are continuous (i.e. domain *n *entirely follows domain *n *- 1 in the sequence). The main objective of these approaches is to identify domain boundary regions. Other methods try to assign residues to particular domains when the domains are discontinuous or split across the sequence (e.g. domain *n *is surrounded by domain *n *- 1 in the sequence). Often these latter methods rely on the availability of accurate 3D models (e.g. modelled by homology), from which the structure is parsed to domains using a 3D to domain parsing algorithm. DOMpro [7] and its server [8] use ranked structural homologues to construct a 3D structure using Modeler [9] then Protein Domain Parser [10] is used to assign the domains. If no homologues are found within a given threshold then ab initio predictions of protein domain boundaries are made from sequence alignments, secondary structure and solvent accessibility predictions. RosettaDom [11] uses many 3D structure models predicted from Rosetta [12] and the Taylor domain parsing algorithm [13].

SnapDragon [14] performs 100 structural predictions from its 3D ab initio system and assigns domains based on an efficient domain parsing algorithm. These methods that rely on 3D structural models are often computationally expensive making them inapplicable for very large scale predictions.

The Domain Guess by Size method [15] guesses domain boundaries solely based on the length distribution of proteins of known structure and is a useful baseline for benchmarking especially ab initio methods. DomSSEA [16] predicts domain boundaries from aligning predicted secondary structure against a database of 3D structures with annotated domain information in the CATH [17] database. Armadillo [18] is also simple and effective – it predicts domain linkers by statistics on the amino acid composition of domain boundaries.

In this paper we concentrate on the evaluation of continuous domain prediction. In other words we are more interested in predicting domain boundaries rather than which domain a residue belongs to. To this end, we ignore the problem of discontinuous domains. Domain boundaries are important features of a protein and have been given particular attention over the years: an analysis of domain boundaries was carried out in [19] with the aim to design boundaries for domain fusion; boundaries are important for inter-domain coupling [20]; altering the length of boundaries connecting domains has been shown to affect protein stability, folding rates and domain-domain orientation [21,22]; ultimately, if the location of protein boundaries is known, barring discontinuous domains, domain identity follows.

Currently well over half of all known protein sequences show some detectable degree of similarity to one or more sequences of known structure. Nearly three quarters of newly deposited structures in the PDB [6] show significant similarity to previously deposited structures [23]. The state of the art predictors at the CASP 6 and 7 competitions [24,25] all contain a template-based component. Homology information is particularly appealing for domain boundary prediction since only some domains for a protein may have homologues while some domains may not, but the boundary can still be inferred by subtracting the homologues from the sequence.

Our method consists of learning boundaries defined by SCOP [26] from evolutionary information in the form of PSI-BLAST [27] sequence alignments, predicted template-based structural information in the form secondary structure [28], solvent accessibility [29], *ϕ *and *ψ *torsion angles [30], contact density [31] and residue-residue contact maps [31]. Along with these features weighted non-gap/gaps in PDB templates and weighted SCOP template definitions are used. All templates are found by simple PSI-BLAST searches on the PDB and SCOP databases. We train 1D Bidirectional Recurrent Neural Networks (BRNN) [32] for the prediction of SCOP defined domain boundaries. The novelty of the method is both in the soft prediction (we do not assume any single piece of information to be true, but rather provide all of them to the RNN) and in the input design, with both SCOP and PDB template profiles used, alongside structural predictions. The structural predictions themselves are made using weighted templates from the PDB with the predictions being significantly better than deriving the information directly from the templates [29].

We show that template information improves over ab initio even for low quality templates, when we design a specialised system for this case. The ab initio predictions compare well with other state-of-the-art ab initio predictors, and the addition of template information always improves over ab initio. As homologues become more accurate predictions are often nearly perfect. It is important to stress that, when homology information is available our algorithm does not take it as the final answer, but rather utilises the homology input in combination with accurate template-based structural information and sequence alignments. This, on average, yields significant improvements over baselines where boundaries are inferred directly from the SCOP homologues.

Although we use simple PSI-BLAST based protocol to find suitable templates, our system is fully modular and may easily incorporate more sophisticated stages with better sensitivity to remote homology (perhaps even by utilising boundary predictions as templates). The method is fast and can be applied to 1000 multi domain proteins in one day on a single 2 GHZ core.

## Methods

Learning domain boundaries consists of mapping *f*(·): ℐ →  where ℐ = (*i*_1_,...,*i*_*N*_) and  = (*o*_1_,...,*o*_*N*_) are the input and output sequences of length *N*. Each *o*_*j *_∈ {0,1} is the output symbol at position *j *resulting in a binary classification problem of domain residues and domain boundary residues. Element *i*_*j *_∈ *I *is the input encoding for position *j *in the sequence. The input encoding is a real numbered vector, *i*_*j *_∈ ℝ^*n*^, where the design choices of *n *and *i*_*j *_largely determines the power of the mapping.

A residue's property at position *j *in the sequence will often depend on local information surrounding *j *and long range information far up and/or down the sequence. We map residues into boundary/non-boundary states by a Bidirectional Recurrent Neural Network (BRNN) [32]:

(1)

where  and  are vectors of hidden states capturing contextual information, respectively, from the left side and right side of the input sequence, and the functions which govern the update of ,  and of the output *o*_*j *_(respectively ,  and ) are realised by Multi-Layered Perceptrons with one hidden layer. *S *in the equations represents the amount of contextual information that is provided explicitly to the  and  networks, or maximum *shortcut *length (see below for more details). The amount of context signal is learned alongside the hidden representation and depends on the error signal produced for a particular protein at a particular residue. This is in contrast to the static window methods where a context window is chosen a priori [33,34] resulting in experiments to determine window sizes using a validation set. In this case danger of overfitting may arise for windows that are too large, especially when the training sets are small. BRNNs are trained by the standard gradient descent algorithm. The gradient of the error (the mutual entropy between target and network output) is computed via an extension of the backpropagation algorithm [35]. BRNNs have been successively applied to many predictive tasks for proteins [28,29,31,32,36,37].

As outputs for individual residues are predicted independently, the raw probabilities of residues being in a domain boundary, *o*_*j*_, contain many local peaks. This is a common problem and has also been reported in [7,38]. In order to mitigate it we use a second stage BRNN that maps the output of the first one into the boundary/non-boundary sequence. The *j*^*th *^input to this second network includes the first-layer predictions in position *j *and first stage predictions averaged over multiple contiguous windows. This input at *j *is the array *I*_*j*_:

(2)

where *k*_*f *_= *j *+ *f*(2*w *+ 1), 2*w *+ 1 is the size of the window over which first-stage predictions are averaged and 2*p *+ 1 is the number of windows considered. In the tests we use *w *= 7 and *p *= 7, as in [28]. Capturing long range dependencies is difficult, especially when using gradient descent [39]. The second stage BRNN described above mitigates this problem, and the presence of shortcut connections (dependencies between a hidden vector and *S *preceding ones with *S *> 1, as in eqn. 1) also helps shortening paths between distant residues. A further way to tackle the problem which we attempt here is similar to that described in [40], and relies on placing shortcut connections over longer ranges, corresponding to predicted contact pairs (see the next section for more details).

### Interaction BRNN

Long ranging information, such as the one usually determining beta-sheets, is difficult to capture using most algorithms. A particular residue, *i*, may be highly coupled with another residue, *j*, far up or down the sequence. A standard BRNN (or, for that, most models we are aware of) fails capture this dependency because of the vanishing gradient problem [39], whereby the gradient of the error rapidly approaches zero as it is propagated backwards through a neural network with multiple layers. An attempt to solve this problem is to place connections into the BRNN between the two residues that are near each other in the three-dimensional space but might span large sequence separations, as for instance in [40]. These interacting connections should allow the model to propagate information (and backpropagate error signals) spanning large sequence separations. Although boundaries are not expected to be coupled with other boundaries this should improve the prediction accuracy of residues interacting within a domain and thus the overall accuracy.

Let us define the estimated probability of contact between residues *i *and *j *as *P*_*i, j*_.

When examining the contacts of residue *j *we look at non-overlapping contiguous windows of contact probabilities up-sequence from *j*:



where *u*_*h *_is an array:



and *kh *= *j *+ *hw*. *w *is the window size over which probabilities are considered, *p *is the number of windows considered, which is the same as the number of shortcut connections. Windows down-sequence, (*d*_-1_,...,*d*_-*p*_), are also taken into account.

We set shortcut connections between all pairs (*j*, *f*_*h*_) such that *f*_*h *_= *argmax*_*y*_*u*_*h*, *y*_, and *f*_-*h *_= *argmax*_*y*_*d*_-*h*, *y*_.

This interaction-based BRNN (IBRNN) takes the form;

(3)

Notice how the the connection strength is multiplied by the probabilities of contact, as estimated by our contact map predictor [41].

In our models we use *w *= 15 and *p *= 5, which means that we connect residue *i *with the one residue over each 15-residue window of the protein that we deem to be most likely to interact with *i*.

### Training, testing set

We start from all chains found in SCOP [26] release 1.73 that are x-ray solved with resolution ≤ 3.0 *Å *and R-factor ≤ 30%. We then use UniqueProt [42] to reduce sequence similarity. We run UniqueProt with options -m custom (those sequences that appear first in the input file are more likely to appear in the output – the sequences are first sorted by decreasing quality), and HSSP [43] distance of 20 (multidomain proteins tend to be longer than 100 amino acids). We leave in boundaries for discontinuous domains, which makes the problem harder than just identifying continuous domain boundaries. In total there are 646 multi domain proteins (set M646) and 321 single domain proteins (S321) in our set. The total number of boundaries is 929.

However, it is important to notice that, since we do not cast the problem as that of mapping a protein into its number of domains, but rather as that of mapping a residue into its boundary vs. non-boundary state, the effective number of examples is the number of residues in the sets (304,221 in total, of which 24,257 boundary residues) rather than the number of proteins, or boundaries. This makes the results of learning quite stable with respect to small variations in initial training conditions or small changes in the architectural parameters of the networks (as observed in preliminary experiments, not shown).

#### PDB and SCOP templates

For each of the proteins in the dataset we search for structural templates in the PDB available on March 25th, 2008 (excluding all entries shorter than 10 residues, leaving 108,076 chains).

**Figure 1:**
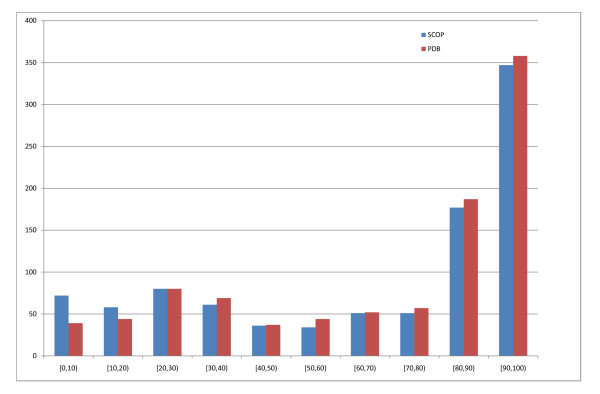
**Best hit distribution**. Distribution of best-hit SCOP (blue) and best-hit PDB (red) sequence identity in the PSI-BLAST templates. Hits above 95% sequence identity excluded.

To generate PDB templates for a protein we run three rounds of PSI-BLAST with parameters *b *= 3000, *e *= 10^-3 ^and *h *= 10^-10 ^against the version of the NR database as available on March 3, 2004 containing over 1.4 million sequences. The NR database is first redundancy reduced at a 98% threshold, leading to a final 1.05 million sequences. We then run a fourth round of PSI-BLAST against the PDB using the PSSM generated in the first three rounds. In this fourth round we use a high expectation parameter (*e *= 10) to include as many hits as possible. We remove from each set of templates all sequences with similarity exceeding 95% between the query and the template to avoid including the query sequence in its own set of templates and to exclude PDB resubmissions of the same structure at different resolution, other chains in N-mers and close homologues. Figure 1 shows the distribution of the templates with this 95% threshold imposed on the sequence identity.

**Figure 2:**
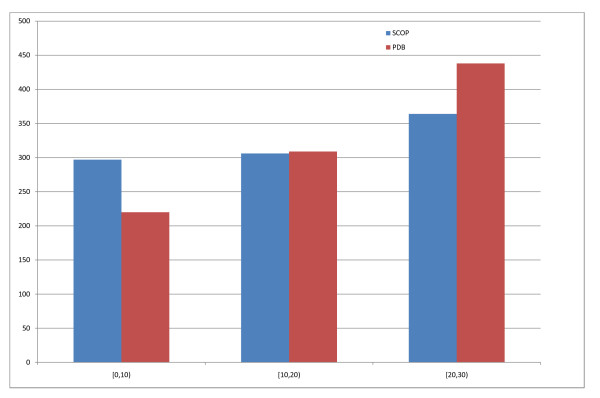
**Best hit distribution, max 25% seq ID allowed**. Distribution of best-hit SCOP (blue) and best-hit PDB (red) sequence identity in the PSI-BLAST templates. Hits above 25% sequence identity excluded.

To train template-based predictions in marginal sequence similarity conditions we create a second set of templates excluding all templates that have a PSI-BLAST hit exceeding 25% sequence identity to the query sequence. To generate SCOP templates we label every PDB template in these two sets with their SCOP defined domain boundaries. We use the 1.73 version of SCOP released in November 2007 which contains 34,494 PDB entries and a total of 97,178 domains. As not all PDB structures have been classified by SCOP the set of SCOP templates is a subset of the PDB templates. Figure 2 shows the distribution of the templates with this 25% threshold imposed on the sequence identity.

### Input design

The input vector at postion *j*,

(4)

contains evolutionary information from multiple sequence alignments , predicted structural features , SCOP templates , and gap information from the PDB templates . The evolutionary profile, , contains 20 units, one for each of the amino acids. The predicted structural features consist of: secondary structure (3 classes), solvent accessibility (4 classes), coarse contact density (4 classes), local structural motifs based on *ϕ *- *ψ *angles (14 classes) (see [30] for a precise definition), and contact maps [29,31]. The structural predictions are based on average weighted PDB templates and sequence information and were shown to be better than simply taken the values directly from the templates [29]. All predictors produce the probability of belonging to a particular structural class and it is these probabilities that are encoded into the  part of the input.

**Figure 3:**
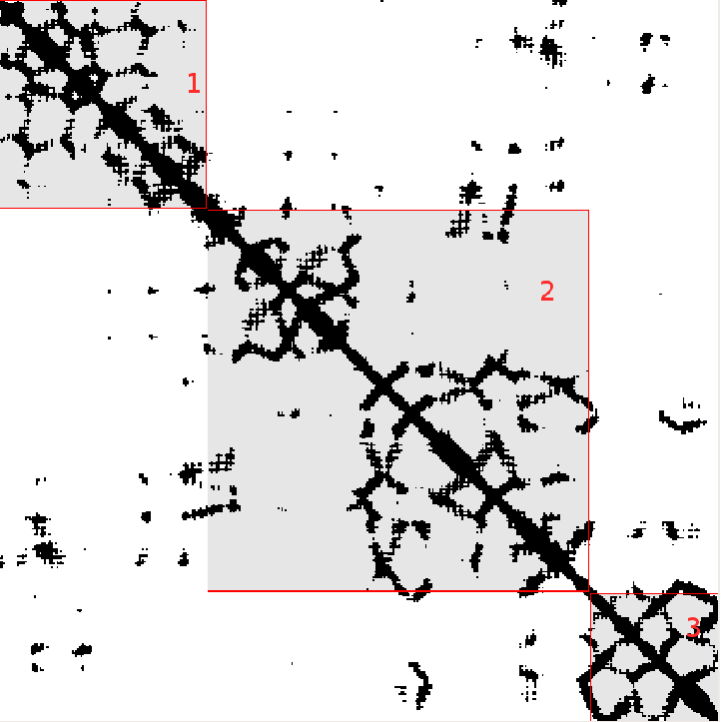
**Multi-Domain Contact Map example**. 13 Å contact map for protein 1KJQA which contains three domains. The three domains are clearly ° distinguishable in the true contact map as the areas with most of the contacting residues pairs. The SCOP definition of the domains for this protein is: domain 1 = residues 1–111, domain 2 = residues 112–317 and domain 3 = residues 318 391. The bounding boxes for each of the domains are labeled. Notice there are a smaller number of contacts that are not part of the domains indicating domain-domain interactions.

**Figure 4:**
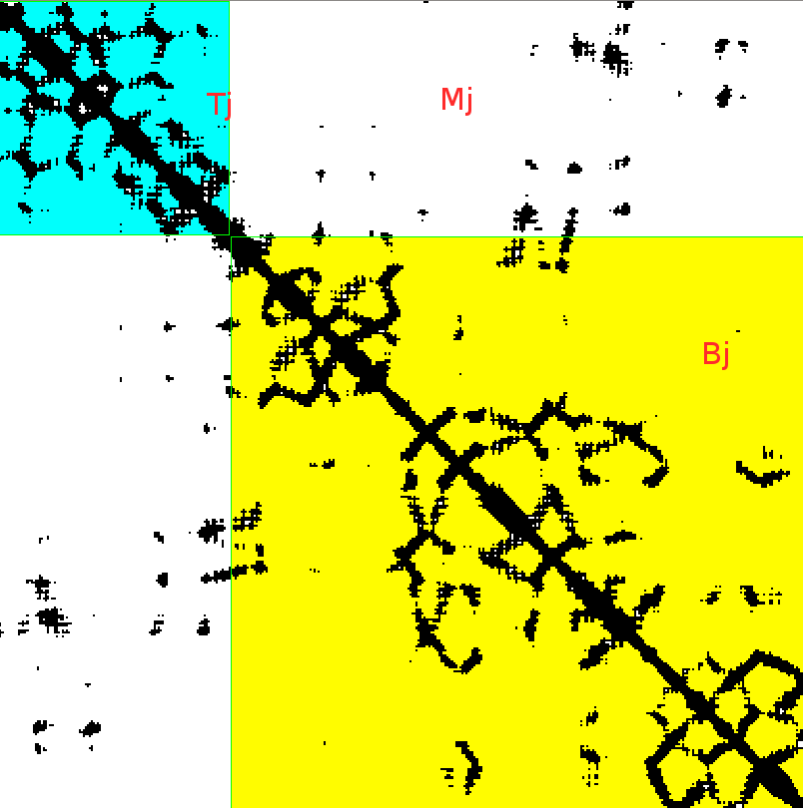
**Multi-Domain Contact Map bounding boxes**. Again protein 1KJQA. The blue, white and yellow areas show the bounding boxes used in the calculations of *T*_*j*_, *M*_*j *_and *B*_*j *_for domain boundary residue 111.

Contact maps should play a special role when predicting domains boundaries. The structurally compact domain regions are clearly distinguishable by visual inspection of a true map as the regions with maximal contact while the boundary regions contain minimal contact (see figure 3 for an example). This observation was exploited in [44] where minimal contact average was determined using covariance analysis on the multiple sequence alignments. Here we derive three numbers which describe contact density in three regions surrounding *j *from maps at a 13 Å threshold:

(5)

where *T*_*j*_, *M*_*j*_, *B*_*j *_correspond to the top left, middle, and bottom right contact/non-contact ratio of the boxes surrounding j – see figure 4. *Cx*, *y *and *NCx*, *y *are the contacts and non-contacts for residue pair (*x*, *y*), where trivial contacts |*x *- *y*| ≤ 3 are ignored. The maps are obtained from a new version of the predictor XXStout [31] which also takes into account template information from the PDB [41].

Ideally a boundary can be identified by large *T*_*j *_and *B*_*j *_and small *M*_*j *_for all *j*. In an initial experiment we found out that local contacting residue pairs are much less informative to determine boundary/non-boundary residues than the global contacting profiles provided by *T*_*j*_, *M*_*j *_and *B*_*j *_(results not shown).

In the results section we show that  with *T*_*j*_, *M*_*j *_and *B*_*j *_improves boundary prediction when the *j *contact maps are sufficiently accurate. The number of units in  is 3 (secondary structure) + 4 (solvent accessibility) + 4 (contact density) + 14 (structural motifs) + 3 (contact maps) = 28.

#### Homology information

Along with structural predictions we input to the network the weighed number of boundaries that we observe in SCOP templates. If *Q *is the total number of templates found for a protein, the first element of the vector  is:

(6)

where *B*_*p *_is equal to one if template number *p *contains a boundary in the position that aligns to the *j*-th residue in the query protein. Note that we extend the original definition of SCOP boundaries by 5 residues towards both termini. If the identity between template *p *and the query is *id*_*p *_and the quality of a template (measured as *X*-ray resolution + R-factor/20, as in [45]) is *q*_*p *_then the weight, *w*_*p*_, is:

(7)

Taking the cube of the identity between template and query allows to drastically reduce the contribution of low-similarity templates when good templates are available. For instance a 90% identity template is weighed two orders of magnitude more than a 20% one. In preliminary tests (not shown) this measure performed better than a number of alternatives. The second and third element of the vector  encode the weighted average coverage and similarity of a column of the template profile as follows:

(8)

where *c*_*p *_is the coverage of the sequence by template *p *(i.e. the fraction of non-gaps in the alignment), and

(9)

Finally weighted gap and non-gap information from the PDB templates used to make the structural predictions are input. These are computed identically to equation 6, 7, 8, and 9 except instead of boundary and non-boundary classes there are gap and non-gap classes. The intuitive reasoning behind  is that domains should be evolutionarily conserved and non-gap values indicate there is a structural fragment in the PDB similar to the query sequence. Both  and  contain 5 units resulting in a total input size of: |*E*| + |*struc*| + |*SCOP*| + |*PDB*| = 20 + 28 + 5 = 5 = 58

#### Measuring performances

To evaluate domain boundary prediction we adopt the domain boundary score used by CASP 6 and 7 [24,25]. A score value is rewarded between any predicted boundary, *P*, and any true boundary, *T*, within eight residues. If *d*_*P*, *T *_is the smallest sequence separation between *P *and *T *(0 in case of any overlap):

(10)

The normalised domain boundary score between all predicted and true domain boundaries is:

(11)

where *np *and *nt *are the total number of predicted domain boundaries and true domain boundaries respectively. Taking the maximum domain boundary count between predicted and true, *max*(*np, nt*), penalises over-prediction and incorporates both sensitivity (precision) and specificity (recall) into one measure. , ensures the closest (predicted vs. true) boundaries are only considered all other values are ignored.

We also consider our performance on single domain proteins, through the F-measure which is the harmonic mean of precision and recall. If *TP *is the number of proteins correctly predicted as single domain, *Pred *is the number of proteins predicted as single domain, and *Obs *is the true number of single domain proteins, recall is  and precision is . Note that template quality, where we refer to it, is always the highest sequence identity between the query and the PDB templates found.

## Results and Discussion

We train and test using a 5-fold cross validation procedure. The following models were trained:

• Ab initio: All structural predictions are made using our ab initio structural prediction servers [46]. In this case we use no contact information, as it led to no improvements in preliminary tests.

• SCOP95: This model takes as input predicted structural information from our template-based structural predictors [29,41], PDB gap/non-gap information and SCOP templates.

• SCOP25: Same as SCOP95 but trained on 25% thresholded templates, i.e. this time no template is allowed that shows more than 25% sequence identity to the query, including to the structural predictors.

• PDB95: This is identical to the SCOP95 models except it now contains no SCOP template information. Note that, although SCOP is a subset of PDB and PDB information is input to this system, it does not include domain boundary annotations.

• PDB25: Same as PDB95 but trained on 25% thresholded templates.

• PDB95_NC and PDB25_NC: Identical to PDB95 and PDB25 except the contact profile in equation 5 is removed.

• IBRNN95: This is identical PDB95 except the BRNN now propagates its information and backpropagates its error along additional shortcut connections that correspond to contacting residue pairs.

• IBRNN25: Same as IBRNN95 but trained on 25% thresholded templates.

All these models have the same architecture, except for extra or missing inputs and are trained by gradient descent. We only ran a small number (less than 10) initial experiments on the sets randomly split in half training and half test to determine a good size for the architecture, while the cross-validations themselves are run only once. Varying the number of parameters of the networks in the initial tests between approximately 5,000 and 10,000 only led to very small changes (at most 0.5%) in predictive quality. When training we place an extra ± 5 residues around the SCOP boundary definitions. However when testing the original SCOP definition is used. Since the problem is extremely imbalanced the optimal threshold (the one that maximises the boundary score, for which see below) for determining boundaries is generally less than 0.5. For this reason we determine the optimal threshold on the training folds and test using this threshold on the test fold.

### 95% distribution

**Figure 5:**
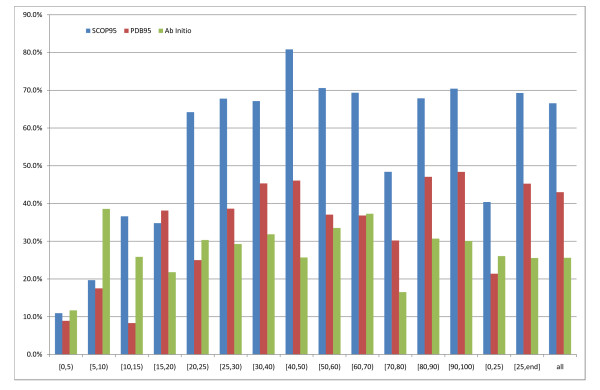
**Comparison between models with 95% max sequence ID templates**. Comparing models across the 95% template distribution. Domain boundary scores as a function of best hit PDB sequence identity. Blue is SCOP95, red is PDB95 and green is ab initio.

Figure 5 shows the domain boundary scores for SCOP95, PDB95 and Ab initio for the 95% template distribution, as a function of template quality. As expected SCOP95 is always clearly better than PDB95 when 20–95% templates are available with differences ranging from 18.2%–39.2%. Overall SCOP95 has a domain boundary score of 66.5% while PDB95 has 43.0%. When only considering templates with similarity greater than 25% these overall values rise to 69.3% and 45.3% respectively. In fact SCOP95 is always better than PDB95 except for a slight decrease in the sequence identity region [15,20)%. When examining the [0,25)% region as a whole we see that SCOP95 has a significantly larger domain boundary score of 40.4% as opposed to an ab initio score of 26.1%. The good performance of SCOP95 in these difficult regions may be due to finding low sequence identity templates where the networks can learn to determine the boundary by subtracting the template from the sequence. Indeed SCOP95 outperforms Ab initio in all template regions above 10% sequence identity. Ab initio is always worse than PDB95 in the [25,95]% similarity region with an overall domain boundary score 19.7% worse, suggesting the BRNN learns to determine boundaries from accurately predicted structural information.

In the [0,25)% region Ab initio is mostly better than PDB95 apart from the [15,20)% interval, for an overall score of 26.1% for Ab initio vs. 21.4% for PDB95. This suggests that PDB templates, and template-based structural predictions are little help when the templates are noisy. However, when a specialised system is built that only learns from noisy templates (see next section), it is still possible to glean enough information from templates to outperform the ab initio predictor. This suggests that, more than the noise itself, the small number of examples in the [0,25)% region is the main reason why PDB95 performs worse than ab initio here.

**Figure 6:**
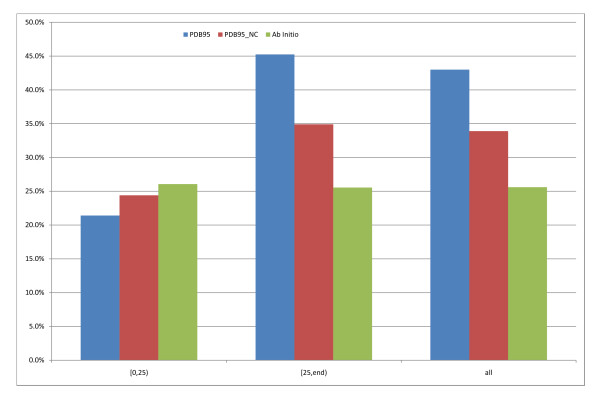
**Comparison between models with 95% max sequence ID templates, with or without contact information**. Comparing the PDB only models with contact information and without. Domain boundary scores as a function of best hit PDB sequence identity. Blue is PDB_95 with contact information, red is PDB_95 without contacts and green is Ab Initio.

In order to assess if contact information improves domain boundary prediction with this template distribution we compare PDB95 with ab initio and an identical version of PDB95 but removing the contact inputs in equation 5 (PDB95_NC). In this case (see figure 6), PDB95_NC performs better than PDB95 (24.4% vs. 21.4%) but still slightly less well than ab initio (26.1%). As expected when templates improve (>25% identity) contact information becomes helpful, leading to significantly better domain boundary location prediction compared to both PDB95_NC and ab initio (PDB95 45.3%, PDB95_NC 34,8%, ab initio 25.6%). This proves that contact information is indeed useful when good quality contact maps are available.

**Figure 7:**
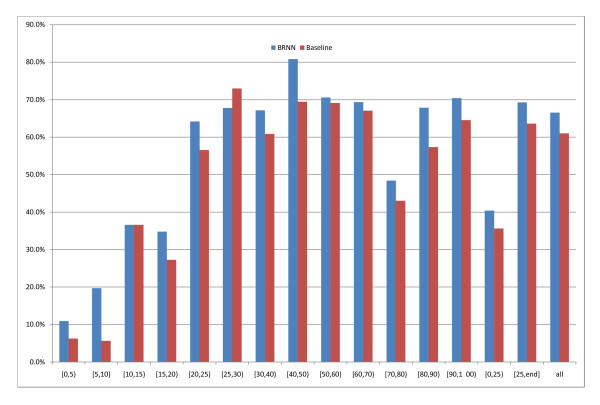
**SCOP95 predictor vs. baseline**. The baseline results (red bins) and SCOP95 results (blue bins) as a function of the identity to the best SCOP template. See text for more details.

**Figure 8:**
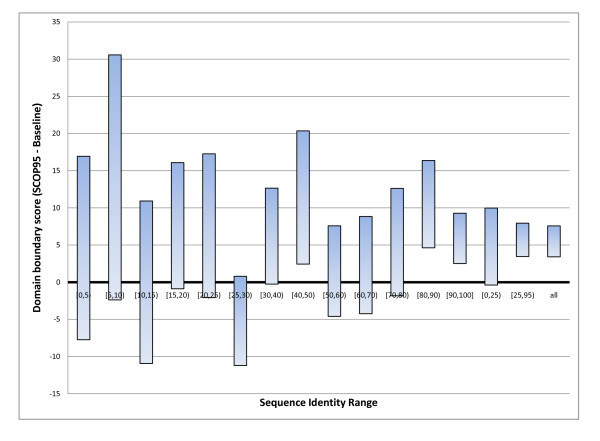
**SCOP95 predictor vs. baseline 2**. The difference between the SCOP95 predictor score and the baseline score (boundaries directly extracted from SCOP templates, as passed as input to the predictor). The size of the blocks represents the error. Although most differences in individual bins are not significant possibly due to the small size of the sample, the overall difference, and difference in the [25,95)% interval are significant, while the gain in the [0,25)% interval is marginal. See text for more details.

Figures 7 and 8 show that our machine learning method, trained in 5-fold cross-validation on the M646 set (see Methods for details), improves over a simple baseline where equation 6 (a weighted average of boundary/non-boundary classes in the templates, normalised between 0 and 1) is adopted as the prediction from the SCOP templates without using any machine learning filtering. Absolute performances are shown in figure 7, while figure 8 focusses on the difference between SCOP95 and the baseline. Preliminary tests showed that this is a better baseline than ones where only the best template or the top ten templates are considered. This is also the same vector provided as input to our system, hence it is a fair baseline to compare the system against as any gains represent enrichment of the information contained in the templates. It is worth noting that the deviations of the absolute results (in Figure 7) of either the baseline or SCOP95 are greater than the deviations of the difference between SCOP95 and baseline on a protein, i.e. the SCOP95 gain is more stable than its absolute score, likely because the variability of the quality of the template is eliminated from the latter (SCOP95 and baseline "see" the same templates). The differences between the prediction and the SCOP baseline are less than 2 standard deviations in all regions of sequence identity to the best template except [40%,50%) and [80%,90%). However the differences are nearly always of the same sign, and overall our system beats the baseline by 5.5%, which is more than 4 standard deviations. The gain in the [25%,100%) area (5.7%) is also more than 4 standard deviations. Encouragingly, in the difficult region (i.e. [0,25)%) there is also a 4.8% improvement over the baseline, although this is marginal, at 1.5 standard deviations.

Finally table 1 shows the F-measures on single domains for all the models trained on the 95% template distribution. In the [0,25)% region ab initio has the best single domain F-measure. Again the SCOP95 model is better by 3.7% at predicting single domain proteins compared to its corresponding baseline. As the templates improve we notice a clear gap between SCOP95 and the PDB only models of PDB95 and PDB95_NC (SCOP95 improves by 9–10%). When there are only PDB templates available ab initio slightly outperforms both PDB95 and PDB95_NC but the larger increase in boundary score outweighs this for [25,95)% templates.

**Table 1:** Single domain F-scores

	SCOP95	Baseline95	PDB95	PDB95_NC	Ab initio
[0,25)%	83.7%	80.0%	85.0%	86.2%	88.1%

[25, end)%	84.9%	84.8%	75.8%	74.5%	77.0%

Single domain F-measures for all models trained with the 95% template distribution.

### 25% distribution

**Figure 9:**
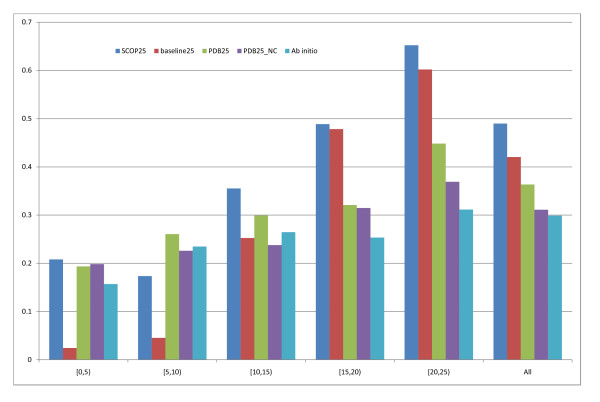
**results for max 25% identity templates**. Results for max 25% identity templates.

In this case we exclude all templates showing a sequence identity greater than 25% to the query. The aim is to build systems that specialise on low-quality templates both by providing more low-quality examples and by not providing any good-quality ones. Figure 9 shows the domain boundary scores for all the models considered in this region. As expected SCOP25 and PDB25 are now always above Ab initio, with a much greater margin and confidence than SCOP95 and PDB95.

However, the F-measure on PDB25 is 8.8% worse than the same model without contact input (PDB25_NC), see table 2. Although the contact profile increases the boundary score, it may lead to over-predicting boundaries. The overall boundary score for the PDB25_NC is 31.1% (PDB95_NC was 24.4% in this region) an increase of 1.3% over ab initio for this region. This coupled with the fact that PDB25_NC has the highest single domain F-measure makes it the best SCOP-less template model for the [0,25)% region. The SCOP based model predicts boundaries with a score of 49% and clearly outperforms its baseline again, on average by 7% (roughly twice the error). Although evaluated on a different distribution of proteins SCOP25 now has a much higher domain boundary score (+8.6%), at the price of a decrease in single domain F-measure (-5.5%).

**Table 2:** Single domain F-scores, max template ID 25%

	SCOP25	Baseline25	PDB25	PDB25_NC	Ab initio
[0,25)%	78.2%	70.4%	70.8%	79.6%	79.1%

Single domain F-measures for all models trained in the 25% template distribution.

### Interaction BRNN

Table 3 shows the overall results when using interaction connections within the BRNN trained with PDB only templates (IBRNN25 and IBRNN95). We can further improve boundary prediction by 3.4% with almost no change in single domain F-measure in the [25,95)% by using the IBRNN. However, the residue-residue contacts are too noisy in the [0,25)% region and therefore single domain F-measure is low compared to other models due to under prediction of boundaries. When training with [0,25)% templates (IBRNN25) both the domain boundary score and single domain F-measure fall by 9% and 5% respectively. Cleary, explicit processing of contacts improves predictions but predicted maps need to be of fair to good quality, especially in order to prevent over-prediction of boundaries and corresponding worsening of single domain predictions.

**Table 3:** IBRNN scores

	IBRNN25	IBRNN95
[25,95)% dbs	-	48.7%(+3.4%)

[25,95)% F	-	75.3%(-0.5%)

[0,25)% dbs	27.3%(-9%)	27.4%(+6%)

[0,25)% F	65.8%(-5%)	77.9%(-7.1%)

IBRNN overall domain boundary scores (dbs) and single domain F-measure (F). In brackets the increase or decrease over the normal BRNN with contact input (i.e. PDB25 and PDB95).

### Comparison with other predictors

**Figure 10:**
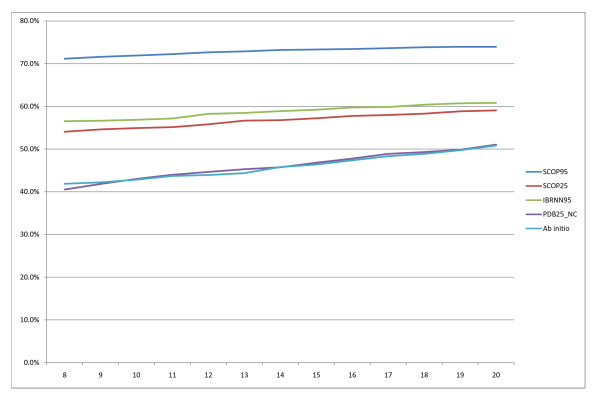
**Recall of domain boundaries**. Recall of domain boundaries as a function of distance from the true boundary.

**Figure 11:**
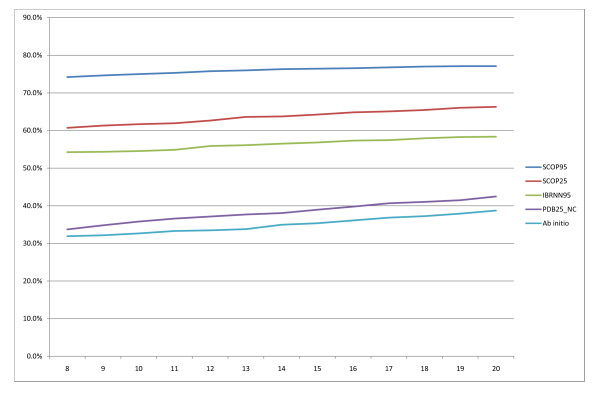
**Precision of domain boundaries**. Precision of domain boundaries as a function of distance from the true boundary.

Comparison of different domain predictors is difficult because previous methods were based on different datasets, domain definitions, benchmarks, cross validations and evaluation procedures. Thus, we take the comparisons made here with caution. State of the art results at CASP 7 have domain boundary scores between 65–69%. Our four best models SCOP95, SCOP25, IBRNN95, PDB25_NC achieve overall domain boundary scores of 66.5%, 48.9%, 46.5% and 31.1%. Figure 10 and 11 show the recall and precision of our models as a function of the distance from the true boundary to consider a prediction a success. Increasing the distance between 8 and 20 results in small improvements in both prediction and recall, slightly more so for the less accurate systems (e.g. Ab initio). It should be noted that this is in essence equivalent to measuring the sensitivity of the results to artificially widening boundary regions.

From the plots we can see that template-based models clearly outperform Ab initio both in the domain boundary score and F-measure on single domain results. At a distance of 8 from the true boundary our recall is 71.2%, 54.1%, 56.5% and 40.5% for the models SCOP95, SCOP25, IBRNN95 and PDB25_NC respectively. The precision of the four models in the same order is 74.2%, 60.7%, 54.2% and 33.7%. The recall and precision (at a distance of 8) of the best server groups at CASP were (all derived from CASP7 assessment plots): DomPro recall 79% and precision 67%, Lee recall 75% and precision 64%, RosettaDom recall 65% and precision 70% and Ginzu 59% recall and 79%. Direct comparisons would not be fair here for two major reasons: we have built SCOP domain predictors, and CASP assignments are normally different from SCOP; especially, while we show that by combining templates and sequence we perform better than by either, we obtain templates by PSI-BLAST, that has much lower sensitivity than many fold recognition components used by the top systems at CASP. However, we have run our methods on the Free Modelling (FM) CASP7 targets (i.e. those for which no suitable templates could be found according to the assessors), allowing only pre-CASP7 templates to be input (as available as of the end of April 2006). Of the 10 FM single domain targets we predict correctly 9 (T0287, T0300, T0307, T0309, T0314, T0319, T0350, T0353, T0361) and one (T0296, the longest one at 445 residues) incorrectly as having two boundaries. As for multi-domain proteins, there was not one single case of a fully FM one. If we focus on multi-domain targets containing at least one domain classified as FM: we predict both boundaries in T0356 (three domains, FM, Template-Based-Modelling, FM) correctly (within 20 residues); we predict the boundary correctly in T0347 (TBM, FM) although we also predict a second spurious boundary; we correctly predict T0316 as being 3-domain and place one of the two boundaries correctly; while we predict T0321's (TBM, TBM/FM) number of domains correctly but boundary location incorrectly by 28 residues.

It should be noted that in none of these cases we find PSI-BLAST templates, so we effectively predict all of them ab initio. CASP's domain assessment also focussed on T0301, which was considered a hard TBM prediction. The assessment article [25] cites one outstanding prediction for this target with an NDO score (Normalized Domain Overlap [25]) of 90 – in this case we correctly predict the protein to be 2-domain for a NDO score of 62.2.

Usually most evaluations in the literature are carried out at a distance ± 20 from the true domain boundary. Our ab initio model has a recall of 50.8% and a precision of 38.7% for domain boundaries within 20 residues of the true boundary. Hence, although this roughly matches the state-of-the-art (see below), in the ab initio case predictions are only of limited practical use. However: for a majority of known protein sequences it is possible to identify a putative homologue in the PDB (for instance, upwards of 80% of queries at the last two CASP competitions have been assessed as template-based); even in the ab initio case it is possible to achieve a higher precision at the cost of reduced recall. For instance we obtain a 55% precision for a recall of 21.2%. Single domains are predicted with a recall of 80.3% and a precision of 78.1% on our dataset. Table 4 shows a summary of some other methods and a short description of the dataset used. Random, is a predictor in which we place the correct number of boundaries within a protein, but in a random position. In this case the Precision/Recall are 24.5%/17.6%, or approximately 2/3 and 1/3 of our ab initio system. ChopNet [47] has a reported boundary recall between 46–51% (when the boundary is within ± 20 of the true boundary) and single domain recall of 73% on their SCOP defined dataset when training on both a CATH and SCOP dataset. When training on a SCOP only dataset as in this study the recall of boundaries seems to be slightly reduced but the single domain recall is drastically reduced to 49%. The ab initio version of DOMAC [7,8] (DomPro at Casp 7) achieves a recall of 88.5% and a precision of 46.5% on single domain proteins, and achieves 27% and 14% recall and precision of domain boundaries within 20 residues, corresponding to an F1 score (the harmonic mean of recall and precision) of 18.4%. The dataset is a balanced, high-quality dataset manually curated by Holland et al. [48]. In order to compare our methods with DOMAC we have also tested our ab initio predictor on this set, and obtain somewhat different recall and precision (16.3% and 19.7%), which yield a similar F1 (17.8%). The Domain Guess by Size algorithm [15] has a recall of 50% for domains shorter than 400 amino acids on a dataset with domain definitions from the Conserved Domain Database [49]. This seems surprisingly good for such a simple method. However predictions were considered correct if a correct prediction is made in one out of top ten predictions, with the accuracy decreasing somewhat when considering the best hit. SnapDragon [14] correctly identifies 47% of its single domain proteins. It also achieves a recall of 42.3% and precision of 39.8% for the boundaries on a mixture of discontinuous and continuous protein domain dataset. The true boundary sizes here were enlarged to a minimum of 21 residues with a correct boundary being ± 10 from this true boundary; making our ± 20 boundary distance comparable. Armadillo [18] achieves a recall of 37% and a precision of 36% on boundaries with a simple amino acid propensity index. Again boundaries were considered correct for ± 20 residues.

**Table 4:** Comparison with other methods

Method	Dataset: number(domain definition)	Recall boundary	Precision boundary	Recall single	Precision single
This study	967(*SCOP*)	50.8%	38.7%	80.3%	78.1%

Random	967(*SCOP*)	17.6%	24.5%	-	-

ChopNet	2127(*SCOP*) + 1300(*CATH*)	46 *- *51%	-	73%	-

DOMAC	156 Holland [48]	27%	14%	88.5	46.5

DGS	1236(*CDD*) [49]	50%	-	-	-

SnapDragon	414(*Taylor*) [13]	42.3%	39.8%	47%	-

Armadillo	585(*CATH *+ *V AST *+ *SCOP*)	37%	36%	-	-

Results for some other methods on their datasets compared to the overall ab initio results in this study.

See text for method citations.

**Figure 12:**
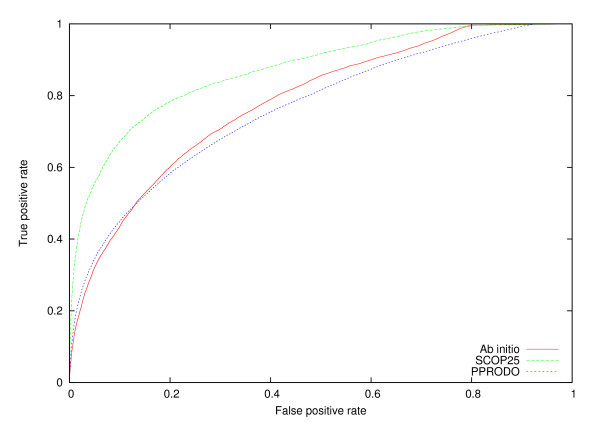
**Ab initio vs. SCOP25 vs. PPRODO**. ROC curves for Ab initio, SCOP25 and PPRODO (see text for more details).

Finally, we directly compare Ab initio and SCOP25 with the predictor in [50] and with PPRODO [51] and report the results in Table 5. In this case the two predictors are optimised for two-domain proteins rather than for a mixture of single and multiple-domain ones. For this reason we test the predictors, where possible, on both our sets and the sets they were optimised on. On the PPRODO set Ab initio roughly matches PPRODO's Recall (64.6% vs. 65.5%) but not Precision (48.3% vs. 65.5%), with slightly more favourable comparisons against [50] that has published Precision and Recall of 62%. On single domain our Recall is similar to PPRODO's. SCOP25 (no templates of any kind are input that show an identity greater than 25% to the query) fares better than Ab initio and roughly equivalently to PPRODO with a Recall/Precision of 69.8%/57.8%. On our sets PPRODO performs quite well, with a Recall/Precision of 56.5%/51.3%, higher than Ab initio (50.8%/38.7%) but this time substantially lower than SCOP25 (59%/66.3%). All systems perform roughly equally well on single domains, with Recalls just over 80%. We were not able to get a version of the CAT dataset also used in [51], and could not obtain a copy of the predictor in [50] so we could not test it on our sets. In figure 12 we report a ROC curve for PPRODO, Ab initio and SCOP25 on our sets. In this case success is measured per residue, rather than per boundary. It is important to notice that we use the original assignment of boundaries adopted by the different programs, i.e. a boundary is extended by 20 residues in both directions to determine positives for PPRODO, and by 5 for Ab initio and SCOP25. In this case the AUC (area under the curve) is 0.76 for PPRODO, 0.78 for Ab initio and 0.87 for SCOP25. If we consider boundaries to be extended by 20 residues, Ab initio and SCOP25 AUC decrease to 0.73 and 0.81, respectively. If we test PPRODO on boundaries extended by 5 residues on both sides, its AUC climbs slightly, to 0.77.

**Table 5:** Comparison with other methods 2

Method	PPRODO sets			M646+S321 sets		
	Recall	Precision	Single	Recall	Precision	Single

Ab initio	64.6%	48.3%	70.0%	50.8%	38.7%	80.3%

SCOP25	69.8%	57.8%	70.2%	59.0%	66.3%	80.0%

PPRODO [51]	65.5%	65.5%	70.2%	56.5%	51.3%	81.9%

[50]	62.0%	62.0%	-	-	-	-

Comparisons with PPRODO [51] and [50].

As our results show, template information, when handled by the best systems (SCOP25, SCOP95, depending on quality) can only improve on the ab initio system for all sequence identity ranges, even in the difficult [0,25)% region. Template-based comparisons are even harder as the data available are more sparse. On a simple two domain set DomSSEA [16] achieves a domain boundary recall of 49% again with ± 20 residues and correctly predicts 82.3% of single domains. DOMAC [8] (DOMAC is the hybrid of DomPro [7] and template based modeling) achieves a domain boundary recall of 50% and a precision of 76.5% (F = 60.5%) within 20 residues of the true domain for its template based part, and an F-measure of 83.7% on single domains, again on the Holland dataset.

Our best template-based system (SCOP95) has boundary recall and precision of 74.0% and 77.1% (F = 75.5%) at ± 20 residues and classifies correctly 85.3% of single domain proteins. Even when we only use marginal templates (SCOP25, max 25%) we achieve boundary recall and precision of 59% and 66.3% (F = 62.4%) and predict 80% of single domain proteins correctly. Although on different sets, all measures are roughly as good as the state-of-the-art systems DomSSEA and DOMAC.

## Conclusion

We have developed a fast system for the prediction of SCOP defined domain boundaries that takes advantage of template-based structural predictions and SCOP templates. Within the limits of comparing systems on different datasets, we have shown that our ab initio system compares well with state-of-the-art ab initio predictors. Our best template-based systems outperform the ab initio system even when poor templates are available, suggesting that not only can they be used for effective domain annotation in the presence of SCOP templates, but they may achieve state-of-the-art performances when only twilight or no templates ([0,25)% sequence identity to the query) are available. We have also shown that our machine learning systems outperform baselines where boundary definitions are extracted directly from the best SCOP template, or from weighed and unweighed profiles of templates. Moreover we have shown that, when high-quality contact maps are factored into the prediction via a sophisticated machine learning model it may be possible to achieve even better results. The systems are entirely automated and can be run on a genome scale on a small cluster of PCs.

Our future work will focus on a number of directions: training and testing our systems on marginal templates, for instance obtained by subtler homology detection algorithms than PSI-BLAST; building a large-scale database of domain predictions to make publicly available, and to feed into the prediction loop alongside SCOP definitions; studying different domain definitions, as for instance those in CATH and PrISM; testing the hypothesis that exon information can lead to improved ab initio predictions [52,53]. Finally, we have set up a public web server implementing the methods we described in this manuscript. The URL of the server is .

## Authors' contributions

IW designed and developed all the predictors, and wrote most of the first draft of the manuscript. AJMM contributed to the design of the homology component. CM produced the final homology detection component and contributed to the manuscript. ER designed an alternative pipeline and provided constant challenges to the development of the final systems. AV assisted many phases of the development and provided useful suggestions. GP sparked the process, supervised all phases, wrote parts of the initial draft, produced the final version of the manuscript, and set up the web server. All authors read and approved the final manuscript.
